# Supplementary material for: Spatial contexts with reliable neural representations support reinstatement of subsequently placed objects
Source: Nat Hum Behav. 2026 Jan 2;10(1):164–81. doi: 10.1038/s41562-025-02379-z (PMC12846921; doi:10.1038/s41562-025-02379-z)
Supplement: Supplementary file 1 — Supplementary Table 1 and Supplementary Figs. 1, 2, 3, 4, 5, 6, 7 and 8. [file 41562_2025_2379_MOESM1_ESM.pdf]

nature human behaviour

Supplementary information https://doi.org/10.1038/s41562-025-02379-z

Spatial contexts with reliable neural representations support reinstatement of subsequently placed objects

In the format provided by the authors and unedited

Supplementary Information

Supplementary Table 1. Virtual memory palace room music 2
Supplementary Figure 1. Example bird's-eye-view drawings 3
Supplementary Figure 2. Relationship between room reliability and room features. 4
Supplementary Figure 3. Additional behavioral measures. 5
Supplementary Figure 4. Perceived object classifier network (POCN) methodology and surface maps. 6
Supplementary Figure 5. Retrieved room classifier network (RRCN) methodology and surface maps. 7
Supplementary Figure 6. Predicting POCN object reinstatement from room reliability. 8
Supplementary Figure 7. Predicting ROCN and POCN object reinstatement from room reliability. 9
Supplementary Figure 8. Correlation plots between room reliability and object reinstatement in ROCN for an example searchlight. 10

Supplementary Table 1. Virtual memory palace room music

Room id	Room Name	Track Name	Track Artist
1	Antiques Room	Rio De Colores	Strunz & Farah
2	TV Room	Birds	Rolando Masís-Obando
3	Candy Room	Liar Liar	The Castaways
4	Classroom	Scatting at the 18th Grammy Awards, Feb. 1976	Ella Fitzgerald & Mel Tormé
5	Tool Room	brazil loops 1	motion_correct
6	Bedroom	Rero	Burne Holiday
7	Floating Islands Room	Brooks Was Here (800% slower; Rolando Masís-Obando StretchMix)	Thomas Newman
8	Planet Room	Electric à la Mass Effect	Rolando Masís-Obando
9	Computer Store Room	Inmersión	Éditus 360
10	Painting Room	Juana y Mi Hermana	Sonámbulo Psicotropical
11	Storage Boxes Room	Aragorn's Speech at the Black Gate	from Lord of the Rings
12	Chess Room	Prelude in G Minor (Op. 23 No. 5)	Sergei Rachmaninoff
13	Empty Room	Wrong Side	Strapping Young Lad
14	Cat Portraits Room	Symphony No. 8 "Unfinished" D 759	Franz Schubert
15	Ruins Room	Memory (Extended)	Nathan Prillaman
16	Clocks Room	Birds Stretched	Rolando Masís-Obando
17	Crystals Room	colorthought – crash _JR 11.4.16	Jakob Reske
18	Colorful Wall Room	Zaggazagga	Rolando Masís-Obando
19	Altar Room	Throatsinging	Rolando Masís-Obando
20	Applecreates Room	Tron Overture (from the Motion Picture TRON: Legacy)	Daft Punk
21	Birthday Party Room	Birthday Horror	Rolando Masís-Obando & the Ken Norman Lab
22	Firepit Room	fire crackles	N/A
23	Human Portraits Room	What Way?	Javier Masís

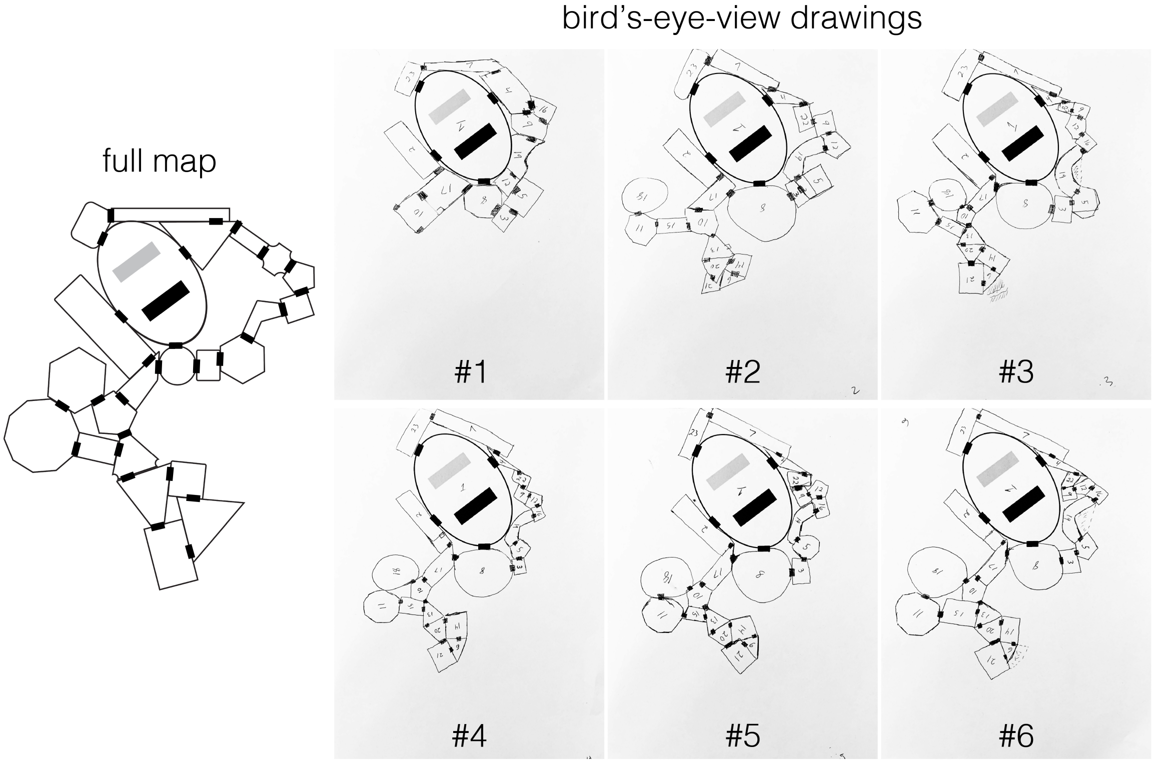

Supplementary Figure 1. Example bird's-eye-view drawings from a single selected participant. Each participant was asked to draw multiple bird's-eye-view maps of the environment across pre- and post-learning sessions to monitor training progress. Each participant was given two sheets of paper, one for the map they had to draw with a central room drawn in, and another with a legend containing the room name and pre-determined room number to facilitate map drawing and room labeling.

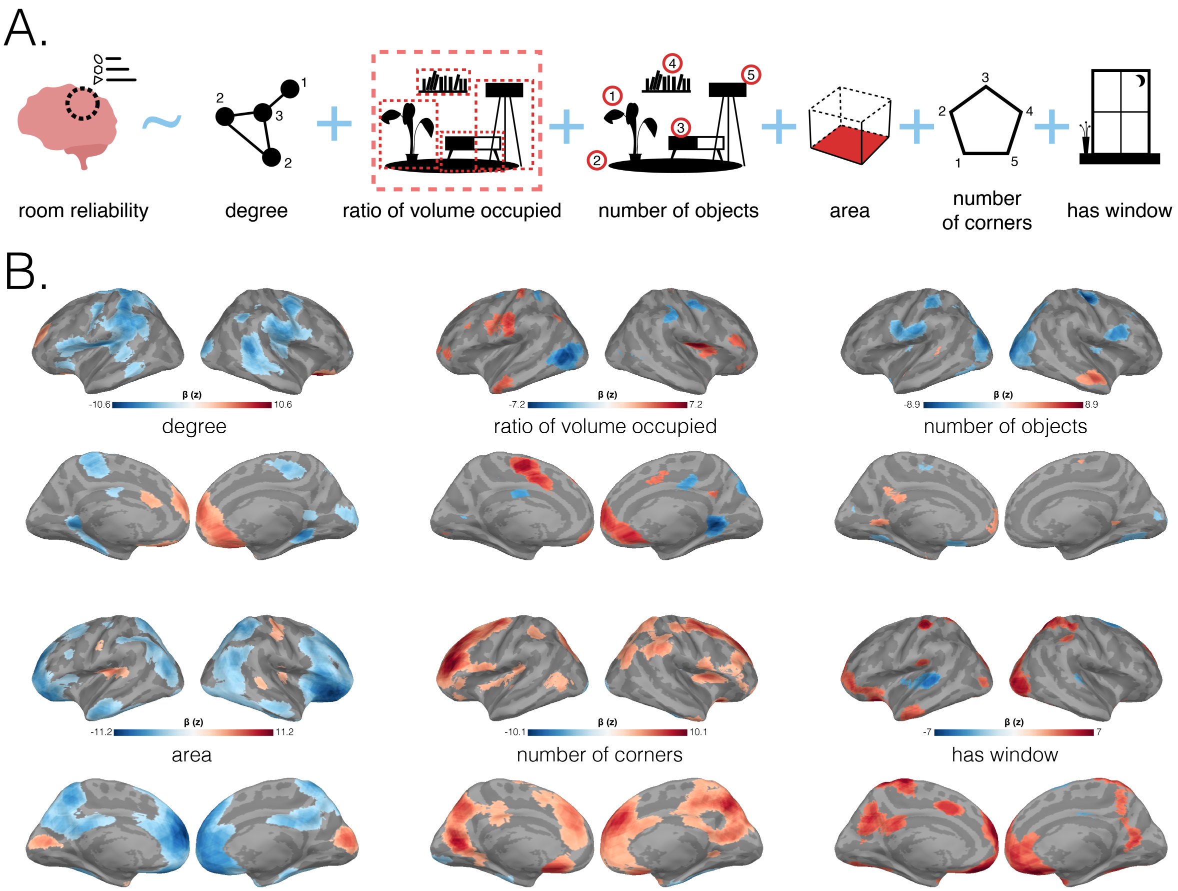

Supplementary Figure 2. Relationship between room reliability and room features. (A.) Regression schematic predicting room reliability with room features. Six different room features were chosen to predict room reliability. From left to right: "degree" (how many rooms are connected to room of interest), "ratio of volume occupied" (the proportion of volume occupied by objects inside a room), "number of objects" (manual count of every object inside a room), "area“ (area covered by room floor), "number of corners“ (sum of wall corners in room), and "has window" (binary, indicating whether this room has a view to the outside). **(B.)** Significant room feature regression coefficients. In a searchlight analysis, reliability for a room (in that searchlight) was predicted by six different room features for each participant. Statistical significance for the resulting beta coefficients was determined by a non-parametric permutation test and FDR-corrected for $q < 0.001$. All six surface maps are colored based on the magnitude of significant z-scored coefficients, with blue showing negative and red showing positive relationships, respectively.

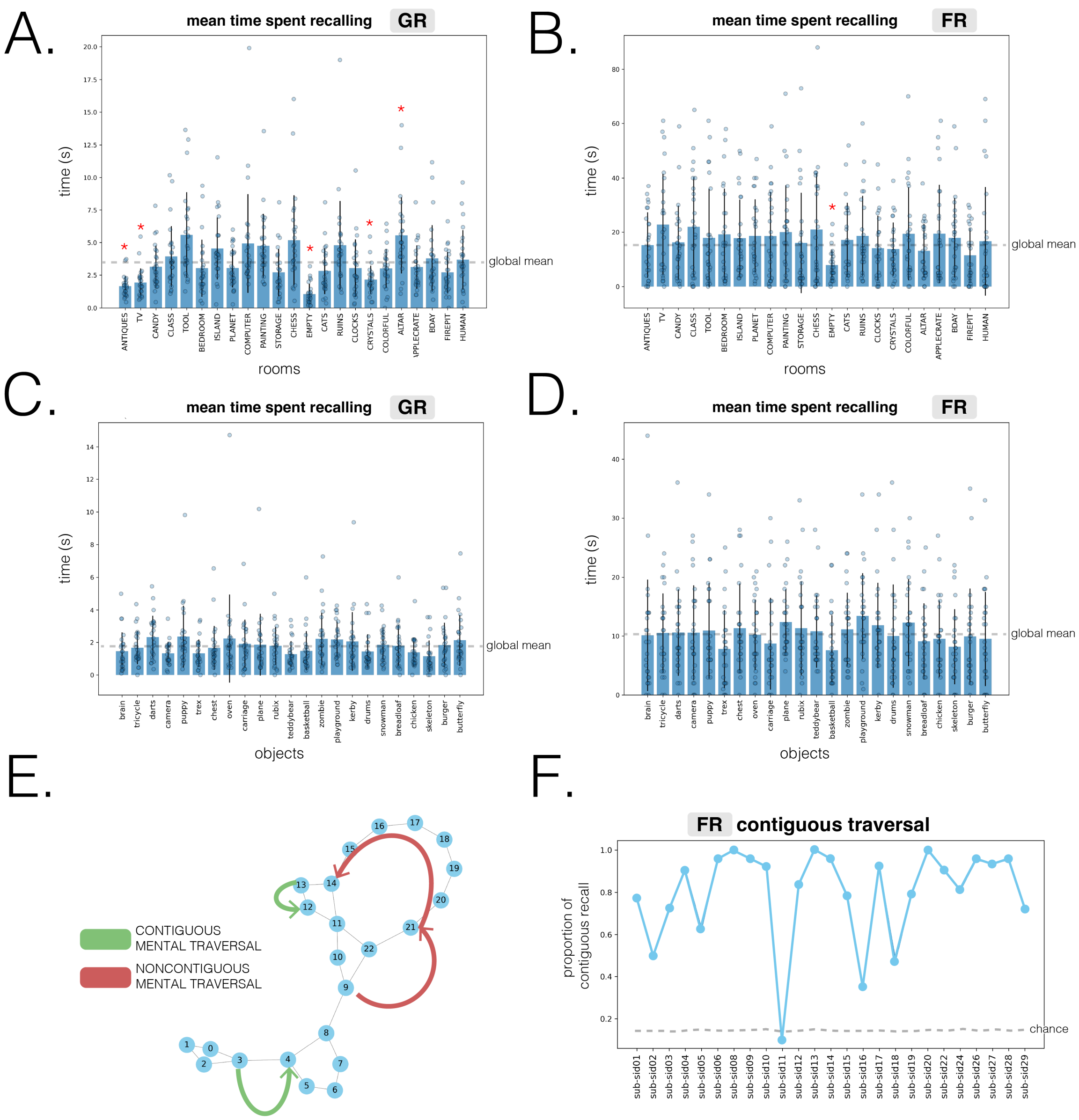

Supplementary Figure 3. Additional behavioral measures. (A–D) Average time participants spent during recall tasks. Statistical testing involved t-tests comparing each item's mean recall time across participants ($n = 25$) against the global mean, with Bonferroni correction across 23 comparisons ($\alpha = 0.05$). **(A.)** Recalling rooms during guided recalls. Significant items after correction were: Antiques room ($t(24) = -11.61$, $p < 0.001$, $d = -2.32$); TV room ($t(24) = -7.08$, $p < 0.001$, $d = -1.42$); Empty room ($t(24) = -15.27$, $p < 0.001$, $d = -3.05$); Crystals room ($t(24) = -6.04$, $p < 0.001$, $d = -1.21$); and Altar room ($t(24) = 3.46$, $p = 0.046$, Cohen's $d = 0.69$). **(B.)** Recalling rooms during free recalls. Empty room ($t(24) = -8.10$, $p < 0.001$, Cohen's $d = -1.62$). **(C.)** Recalling objects during guided recalls. **(D.)** Recalling objects during free recalls. For **A-D**, bar charts describe mean recall time across participants, with error bars indicating standard deviation, dots indicating participant means, and dashed gray lines indicating the global mean. Red stars above bar plots indicate a significant difference in time from the global mean, for Bonferroni corrected $p < 0.05$. **(E.)** Contiguous vs noncontiguous mental traversal. When recalling rooms explored in VR, participants either recalled spatially adjacent (contiguous) rooms or jumped between rooms separated by more than one degree (noncontiguous). **(F.)** Contiguity in free recall. We measured the proportion of contiguous room transitions during free recall by computing, for each participant, the proportion of successive recalls that involved spatially adjacent rooms. We then compared these proportions against chance levels using a paired t-test. Across participants ($n = 25$), spatially adjacent rooms were recalled significantly more often than expected by chance ($t(24) = 14.19$, $p < 0.001$, Cohen's $d_z = 2.99$), indicating an unprompted bias toward contiguous mental traversal.

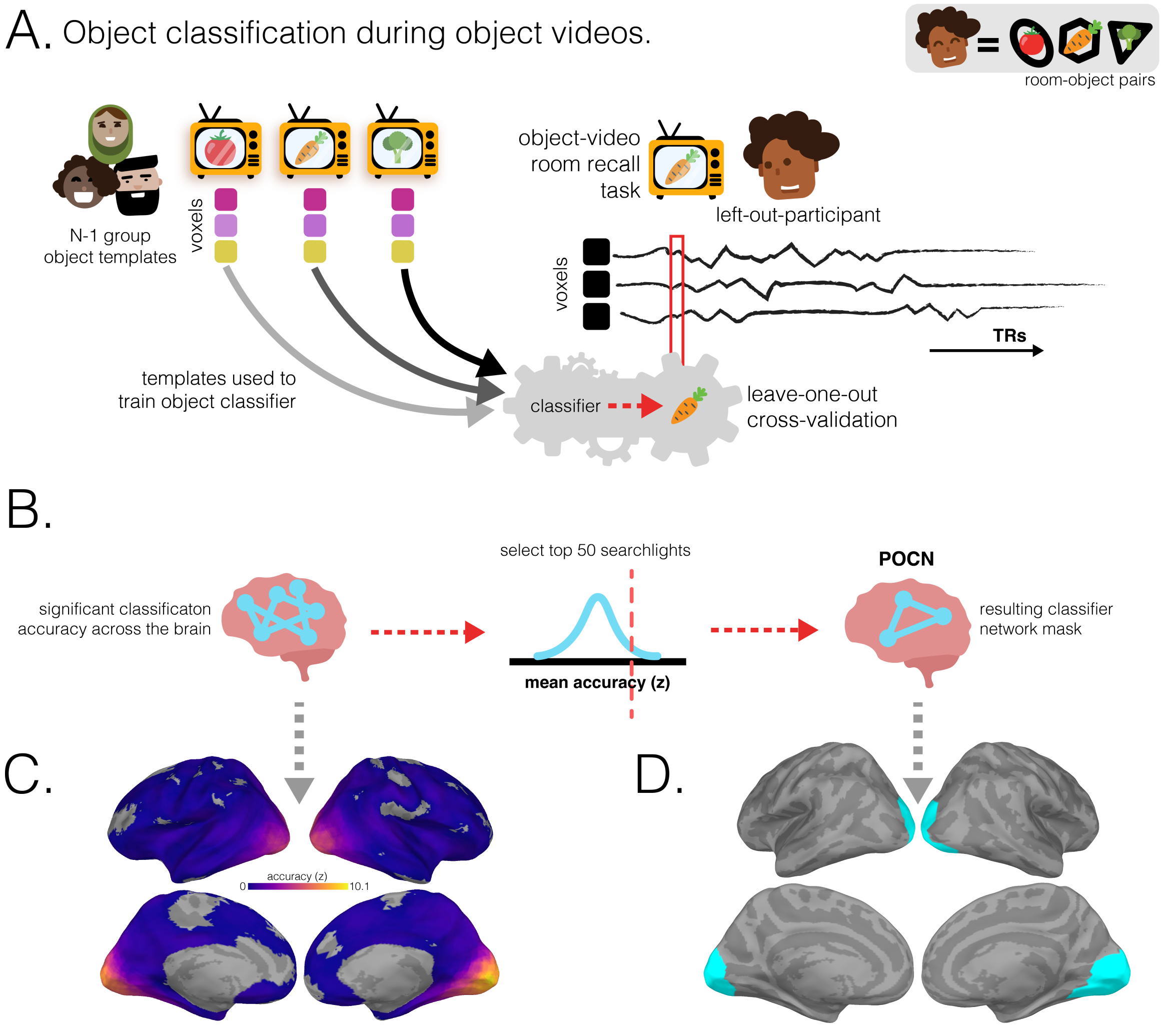

Supplementary Figure 4. Perceived object classifier network (POCN) methodology and surface maps. (A.) As for the ROCN, the characteristic object patterns of the N-1 group were used to train a multinomial logistic classifier. Here, this classifier was applied to timepoints when the left-out participant was viewing objects during the post-learning object videos, e.g. viewing a video of a carrot in this example. We then measured the fraction of timepoints during the object video that were classified as activating the carrot representation. **(B.)** After object classification was performed for both post-learning object video runs for each participant, average classification accuracies across participants for each searchlight were then averaged across both runs and then z-scored relative to a null distribution. The top 50 searchlights were then selected to form the POCN. **(C.)** Average object classification accuracy during object encoding (thresholded to show searchlights with above-chance accuracy). **(D.)** Perceived Object Classifier Network. The surface map shows the top 50 best object classifying searchlights during both post-learning object video tasks across participants.

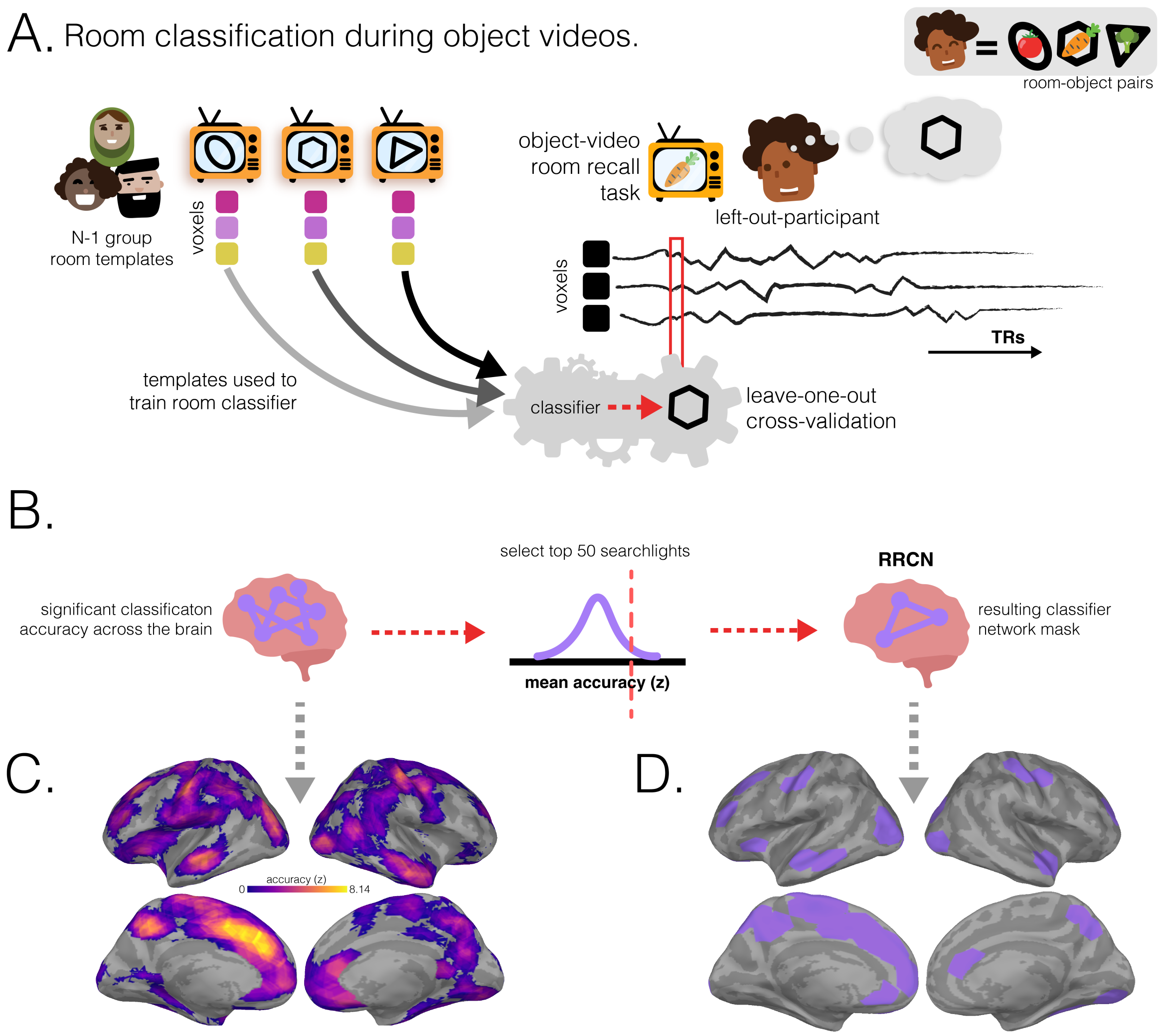

Supplementary Figure 5. Retrieved room classifier network (RRCN) methodology and surface maps. (A.) The characteristic room patterns of the N-1 group – evoked during a separate phase of the study in which participants viewed room videos before learning room-object associations – were used to train a multinomial logistic classifier. This classifier was then applied to each timepoint on the left-out participant's object-video room recall data. In the pictured example, the left-out participant, Fernando, is recalling the hexagon room that was paired with the carrot object currently being presented. The room classifier, trained on patterns evoked when other participants viewed the rooms, was applied to each timepoint of Fernando's carrot viewing. We then measured the fraction of timepoints during the object video that were classified as activating the hexagon representation. **(B.)** After room classification was performed for both post-learning object video runs for each participant, average classification accuracies across participants for each searchlight were then averaged across both runs and then z-scored relative to a null distribution. The top 50 searchlights were then selected to form the RRCN. **(C.)** Average object classification accuracy during object encoding (thresholded to show searchlights with above-chance accuracy). **(D.)** Retrieved Room Classifier Network. The surface map shows the top 50 best room classifying searchlights during both post-learning object video tasks across participants.

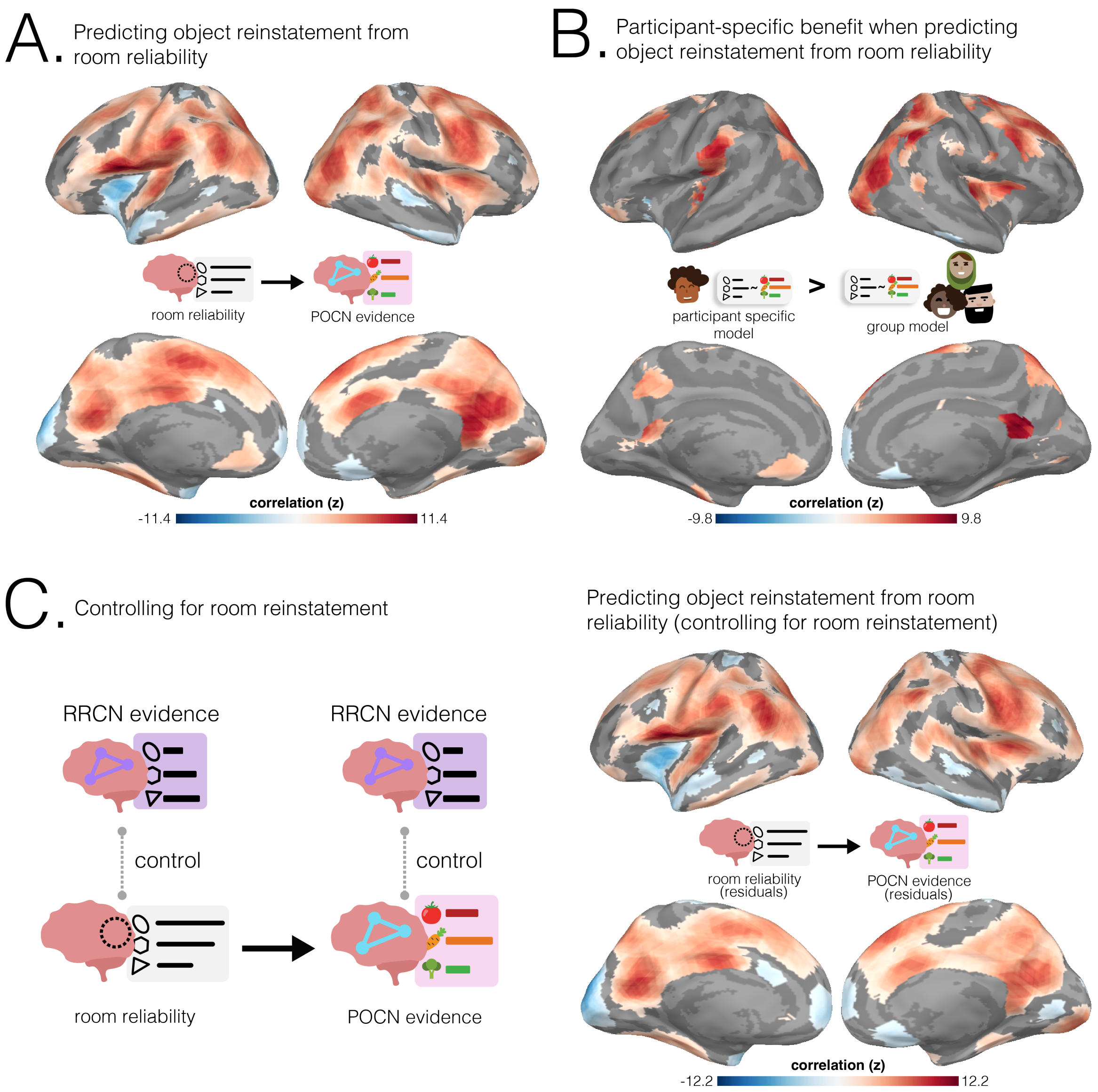

Supplementary Figure 6. Predicting POCN object reinstatement from room reliability. (A.) Relationship between POCN object reinstatement and room reliability. Regions where room reliability predicted POCN object reinstatement across both guided and free recalls. Objects placed in rooms with the most pre-learning neural stability in these regions were reinstated the most strongly during retrieval. **(B.)** Model comparison results. Regions where room reliability predicted POCN object reinstatement across both guided and free recalls and there was a predictive benefit from participant-specific room reliability. In these regions, the rooms that were most reliable for a specific participant (rather than rooms that were generally reliable across the group) were predictive of object recall for that specific participant. The surface maps presented in **B** show the intersection of the participant-specific models shown in **A** and the regions where there was a significant positive difference in the coefficient of determination between the original participant-specific model and the N-1 group model. Statistical significance for the differences between the coefficients of determination was determined by comparing the differences to a null distribution and FDR-correcting for $q < 0.05$. **(C.)** Controlling for room reinstatement. Left column: Schematic illustrating how room reinstatement evidence in RRCN (during timepoints in which participants verbally recalled a room or its paired-object) was regressed out of room reliability and POCN object reinstatement scores. Room reliability residuals were then correlated at each searchlight with POCN object reinstatement residuals. Right column: Regions where room reliability predicted POCN object reinstatement after controlling for room reinstatement during room and object recall. The surface maps presented in **A** and **C** were statistically thresholded by comparing correlations to a null distribution and then FDR-correcting for $q < 0.05$. All three surface maps are colored based on the magnitude of the z-scored correlation values of the participant-specific model, with blue showing negative and red showing positive relationships, respectively.

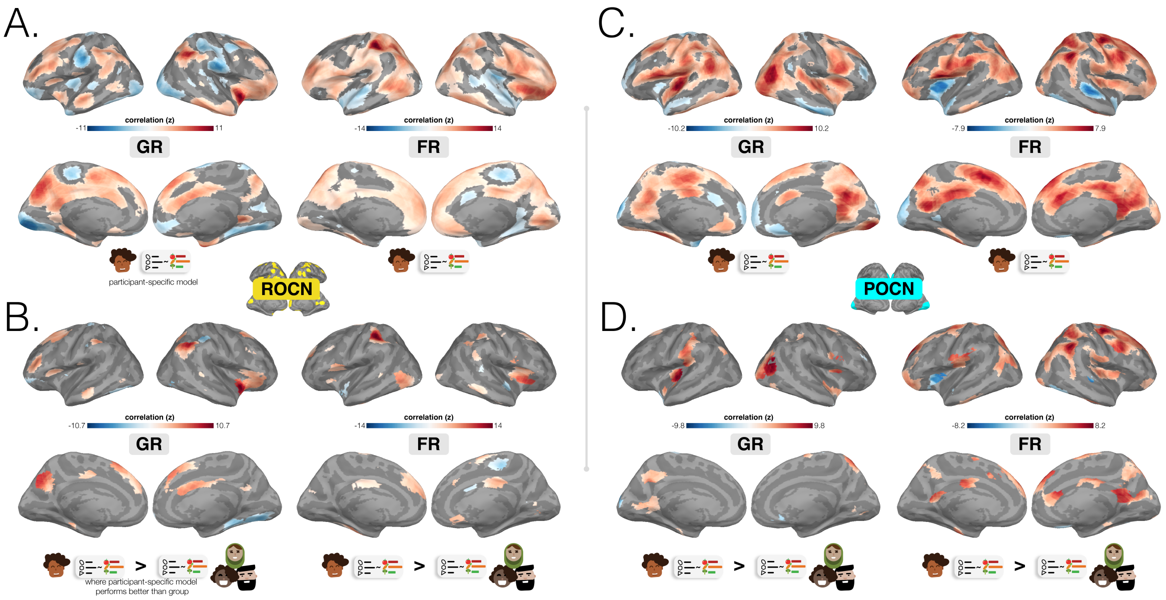

Supplementary Figure 7. Predicting ROCN and POCN object reinstatement from room reliability. Relationships between room reliability and classifier network object reinstatement evidence. ROCN and POCN results are shown to the left and right of the dividing gray line, respectively. **(A.)** Regions where room reliability predicted ROCN object reinstatement in guided recalls (GR; left) and free recalls (FR; right). **(B.)** Model comparison results. Regions where room reliability predicted ROCN object reinstatement and there was a predictive benefit from participant-specific room reliability, shown separately for guided and free recalls. **(C.)** Regions where room reliability predicted POCN object reinstatement in guided recalls (GR; left) and free recalls (FR; right). **(D.)** Model comparison results. Regions where room reliability predicted POCN object reinstatement and there was a predictive benefit from participant-specific room reliability, shown separately for guided and free recalls. The surface maps presented in A and C were statistically thresholded by comparing correlations to a null distribution and then FDR-correcting for $q < 0.05$. The surface maps presented in **B** and **D** show the intersection of the participant-specific models (**A** and **C**) and the regions where there was a significant positive difference in the coefficient of determination between the original participant-specific model and the N-1 group model. Statistical significance for the differences between the coefficients of determination was determined by comparing the differences to a null distribution and FDR-correcting for $q < 0.05$. All surface maps are colored based on the magnitude of the z-scored correlation values of the participant-specific model, with blue showing negative and red showing positive relationships, respectively.

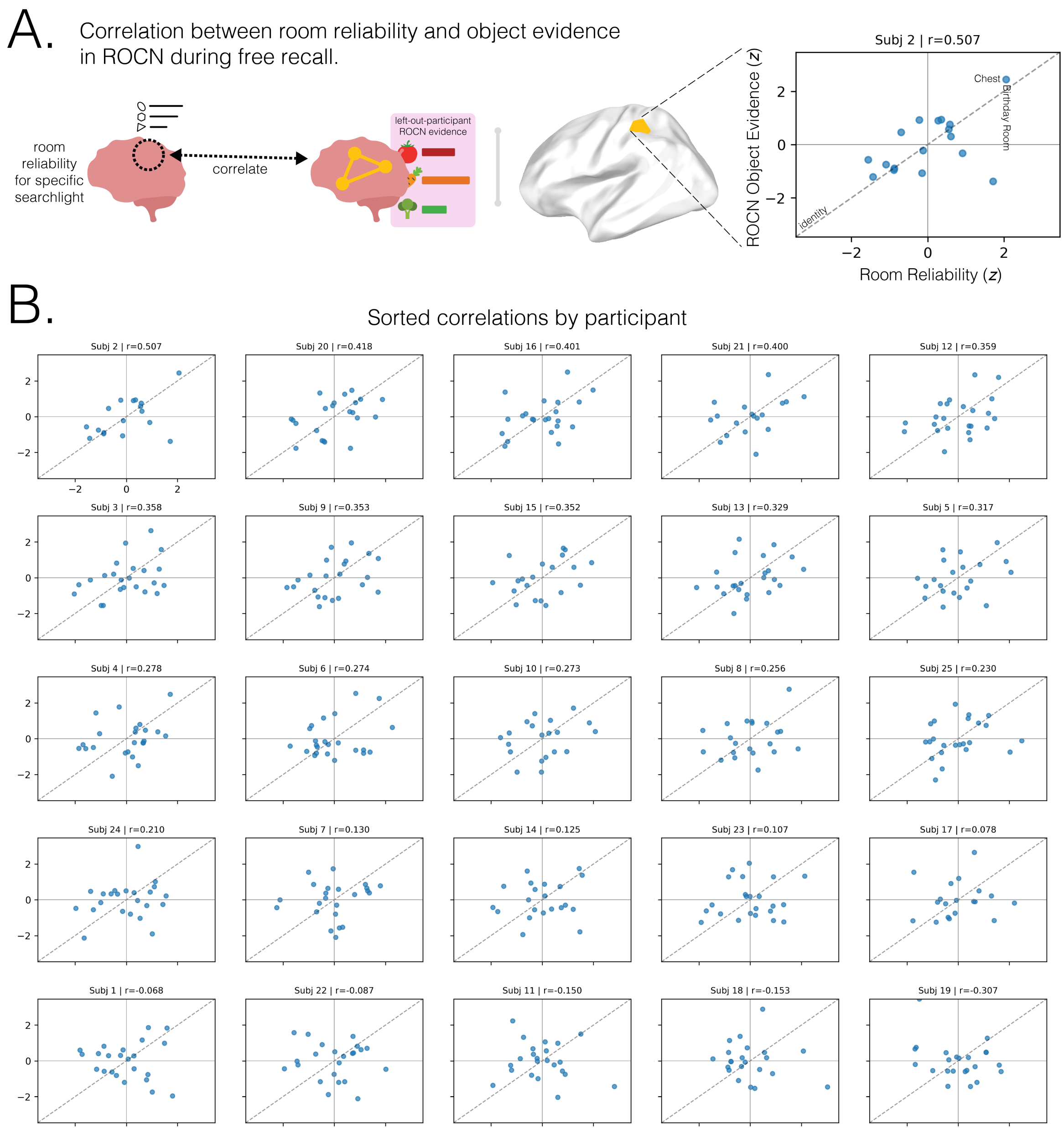

Supplementary Figure 8. Correlation plots between room reliability and object reinstatement in ROCN for an example searchlight. **(A.)** Scatterplot illustrating the correlation for an example searchlight and participant. For each participant's free recall (FR) data, we computed the correlation between room reliability and ROCN object reinstatement evidence in the ROCN mask. **(B.)** Individual scatterplots for all 25 participants showing the relationship between room reliability scores and ROCN reinstatement evidence, extracted from the example searchlight seen in **A**. Plots are sorted by participants, moving from left to right in order of highest to lowest correlations, respectively. Dashed lines in scatterplots represent the identity line ($y = x$), and Pearson's r correlation values are reported on the title-line of each plot. Room reliability and ROCN object reinstatement scores were z-scored to facilitate visualization of relationships.
