# Supplementary material for: Spatial contexts with reliable neural representations support reinstatement of subsequently placed objects
Source: Nat Hum Behav. 2026 Jan 2;10(1):164–81. doi: 10.1038/s41562-025-02379-z (PMC12846921; doi:10.1038/s41562-025-02379-z)
Supplement: Supplementary file 2 — Reporting Summary [file 41562_2025_2379_MOESM2_ESM.pdf]

nature portfolio

Corresponding author(s): Rolando Masís-Obando

Last updated by author(s): 2025/11/04

Reporting Summary

Nature Portfolio wishes to improve the reproducibility of the work that we publish. This form provides structure for consistency and transparency in reporting. For further information on Nature Portfolio policies, see our Editorial Policies and the Editorial Policy Checklist.

Statistics

For all statistical analyses, confirm that the following items are present in the figure legend, table legend, main text, or Methods section.

n/a	Confirmed	
☐	☒	The exact sample size (n) for each experimental group/condition, given as a discrete number and unit of measurement
☐	☒	A statement on whether measurements were taken from distinct samples or whether the same sample was measured repeatedly
☐	☒	The statistical test(s) used AND whether they are one- or two-sided<br>*Only common tests should be described solely by name; describe more complex techniques in the Methods section.*
☐	☒	A description of all covariates tested
☐	☒	A description of any assumptions or corrections, such as tests of normality and adjustment for multiple comparisons
☐	☒	A full description of the statistical parameters including central tendency (e.g. means) or other basic estimates (e.g. regression coefficient) AND variation (e.g. standard deviation) or associated estimates of uncertainty (e.g. confidence intervals)
☐	☒	For null hypothesis testing, the test statistic (e.g. F, t, r) with confidence intervals, effect sizes, degrees of freedom and P value noted<br>*Give P values as exact values whenever suitable.*
☒	☐	For Bayesian analysis, information on the choice of priors and Markov chain Monte Carlo settings
☐	☒	For hierarchical and complex designs, identification of the appropriate level for tests and full reporting of outcomes
☐	☒	Estimates of effect sizes (e.g. Cohen's d, Pearson's r), indicating how they were calculated

Our web collection on statistics for biologists contains articles on many of the points above.

Software and code

Policy information about availability of computer code

Data collection	Unity 3D (5.5.2f1 & 2017.1.2f1) for virtual environment creation and Virtual Reality Toolkit (VRTK), a virtual-reality programming tool-kit for Unity3D, for participant VR interactivity. PsychoPy for task presentation in the scanner, OBS Studio for video capture. Additional software used for 3D modeling included SketchUp and Blender.
Data analysis	Python 3.x (NumPy, SciPy, scikit-learn, MNE-Python), fMRIPrep v1.0.3 (Nipype-based), FreeSurfer v6.0.0, ANTs v2.1.0, FSL v5.0.9, AFNI v16.2.07. Visualization with Python and MNE-Python. Participant recalls were minimally transcribed using UPenn's TotalRecall. All analysis scripts are publicly available on GitHub: https://github.com/rmasiso/MemoryPalaceReliability -- custom algorithms (e.g., searchlight pattern reliability and reinstatement analyses) are described in the Methods and provided in the repository.

For manuscripts utilizing custom algorithms or software that are central to the research but not yet described in published literature, software must be made available to editors and reviewers. We strongly encourage code deposition in a community repository (e.g. GitHub). See the Nature Portfolio guidelines for submitting code & software for further information.

Data

Policy information about availability of data

All manuscripts must include a data availability statement. This statement should provide the following information, where applicable:

- Accession codes, unique identifiers, or web links for publicly available datasets
- A description of any restrictions on data availability
- For clinical datasets or third party data, please ensure that the statement adheres to our policy

All raw and preprocessed MRI and behavioral data are openly available on OpenNeuro: https://openneuro.org/datasets/ds005704

Research involving human participants, their data, or biological material

Policy information about studies with human participants or human data. See also policy information about sex, gender (identity/presentation), and sexual orientation and race, ethnicity and racism.

Reporting on sex and gender	Both male and female participants were included in approximately equal numbers (16 females, 14 males initially; final analyzed sample: 11 females, 14 males).<br>Sex was recorded through participant self-report at enrollment and used solely for demographic description; no analyses were stratified or compared by sex or gender identity, as the study's hypotheses did not pertain to sex-related differences.
Reporting on race, ethnicity, or other socially relevant groupings	Participants were recruited from the Princeton University community and surrounding area.<br>Recruitment aimed to ensure that underrepresented minorities were included in approximate proportion to the local population.<br>Race or ethnicity were not collected as experimental variables and were not included in any analyses, as the study objectives concerned general principles of spatial memory and neural reinstatement.
Population characteristics	Healthy adults aged 21–32 years, with normal or corrected-to-normal vision, and no reported neurological or psychiatric disorders.<br>All participants were fluent in English and had prior experience using computers or gaming interfaces.<br>The final analyzed sample comprised 25 participants (11 female, 14 male).
Recruitment	Participants were recruited through the Princeton University research participation system, local flyers, and online postings.<br>Eligibility criteria included normal or corrected-to-normal vision and no history of neurological or psychiatric illness.<br>Recruitment aimed to balance gender representation and reflect the demographic composition of the local community.<br>Participants provided written informed consent and received monetary compensation.
Ethics oversight	All procedures were approved by the Princeton University Institutional Review Board (IRB #7225).<br>All participants provided written informed consent prior to participation and were debriefed following the experiment.<br>The study complied with all relevant ethical regulations for research involving human participants.

Note that full information on the approval of the study protocol must also be provided in the manuscript.

Field-specific reporting

Please select the one below that is the best fit for your research. If you are not sure, read the appropriate sections before making your selection.

☐ Life sciences ☒ Behavioural & social sciences ☐ Ecological, evolutionary & environmental sciences

For a reference copy of the document with all sections, see nature.com/documents/nr-reporting-summary-flat.pdf

Behavioural & social sciences study design

All studies must disclose on these points even when the disclosure is negative.

Study description	This study investigated how reliable neural representations of spatial contexts (rooms) support memory reinstatement of associated objects. Participants learned a 23-room virtual environment in virtual reality (VR), after being scanned for neural snapshots of the rooms, they returned to VR to learn newly placed objects in each room, and then recalled the rooms and objects during fMRI scanning. The research combined immersive behavioral learning (in VR) with whole-brain fMRI analyses to link stable room representations ("spatial context reliability") to subsequent object reinstatement.
Research sample	Thirty healthy adults aged 21–32 years (16 female, 14 male) participated. All had normal or corrected-to-normal vision and no history of neurological or psychiatric conditions. Five participants were excluded due to missing or corrupted MRI data, leaving n = 25 for analysis.
Sampling strategy	Participants were recruited from the Princeton University community and surrounding area through flyers, mailing lists, and online postings. The target sample size was based on prior fMRI memory and reinstatement studies using similar within-subject designs (typically 20–30 participants), providing sufficient power for searchlight and cross-participant analyses. Recruitment aimed to balance

gender representation and reflect local demographic diversity.

Data collection	Behavioral and VR data were collected in the Princeton Neuroscience Institute behavioral labs; MRI data were collected on a Siemens Prisma 3 T scanner with a 64-channel head coil.<br>Virtual reality tasks were implemented in Unity 3D using custom code and the Virtual Reality Toolkit (VRTK). Stimuli were presented in the scanner via PsychoPy. Speech recall data were recorded with the scanner's integrated microphone and annotated using TotalRecall. Data were preprocessed using fMRIPrep, FreeSurfer, FSL, AFNI, ANTs, and custom Python scripts.
Timing	Each participant completed the experiment across two consecutive days:<br><br>Day 1: Behavioral VR learning and map-drawing sessions (~1.5 hours).<br><br>Day 2: Two fMRI scanning sessions (pre-learning and post-learning, ~3 hours total) and an intermediate VR session (~0.5 hours).<br>Data collection occurred between 2018 and 2019 at Princeton University.
Data exclusions	Five participants were excluded due to missing or corrupted MRI files that prevented preprocessing and analysis.<br>No exclusions were made based on demographic characteristics. All other participants (n = 25) were included in every reported analysis.
Non-participation	All enrolled participants completed the full experimental protocol across both days, except those excluded due to technical data loss.<br>No participant withdrew voluntarily.
Randomization	Room–object pairings were randomized independently for each participant to prevent systematic associations between specific rooms and objects.<br>The order of room and object videos during scanning was pseudorandomized within task blocks.<br>Classifier analyses used leave-one-participant-out cross-validation to eliminate circularity and ensure unbiased generalization across subjects.

Reporting for specific materials, systems and methods

We require information from authors about some types of materials, experimental systems and methods used in many studies. Here, indicate whether each material, system or method listed is relevant to your study. If you are not sure if a list item applies to your research, read the appropriate section before selecting a response.

Materials & experimental systems

n/a	Involved in the study	
☒	☐	Antibodies
☒	☐	Eukaryotic cell lines
☒	☐	Palaeontology and archaeology
☒	☐	Animals and other organisms
☒	☐	Clinical data
☒	☐	Dual use research of concern
☒	☐	Plants

Methods

n/a	Involved in the study	
☒	☐	ChIP-seq
☒	☐	Flow cytometry
☐	☒	MRI-based neuroimaging

Plants

Seed stocks	Plants were not used in this study.
Novel plant genotypes	Plants were not used in this study.
Authentication	Plants were not used in this study.

Magnetic resonance imaging

Experimental design

Design type	Within-subjects, cross-participant design. Participants completed both behavioral (VR) and fMRI tasks across two consecutive days. Day 1 focused on spatial learning in VR; Day 2 included pre- and post-learning fMRI scans as well as a short VR task in which participants had to learn the newly placed objects in the virtual environment rooms.

Design specifications	All participants underwent identical task sequences (VR environment learning, pre-learning scan, VR newly placed objects learning, and post-learning scan).<br>Stimulus order within each scanning task was pseudorandomized.<br>Classifier analyses used leave-one-participant-out cross-validation.
Behavioral performance measures	VR foraging scores, map-drawing accuracy, verbal recall duration, and recall contiguity were logged and analyzed for learning progression and memory retrieval structure.

Acquisition

Imaging type(s)	Functional and structural MRI (BOLD EPI and T1-weighted anatomical imaging).
Field strength	3 Tesla (Siemens Prisma scanner, 64-channel head coil).
Sequence & imaging parameters	Multiband EPI sequence (TR = 1300 ms, TE = 33 ms, flip angle = 80°)<br>Slice thickness: 2 mm<br>Field of view: 192 mm²<br>Multiband factor (SMS) = 4<br>Whole-brain coverage<br>T1-weighted anatomical: MPRAGE, 1 mm³ isotropic resolution
Area of acquisition	Whole brain, including cortical and subcortical regions.

Diffusion MRI ☐ Used ☒ Not used

Preprocessing

Preprocessing software	fMRIPrep 1.0.3 (Nipype-based pipeline)<br>FreeSurfer 6.0.0 for surface reconstruction<br>ANTs 2.1.0, FSL 5.0.9, AFNI 16.2.07 for motion correction and registration<br>Custom Python scripts (NumPy, SciPy, MNE-Python) for nuisance regression and z-scoring
Normalization	Surface- and volume-based normalization using ANTs nonlinear registration.
Normalization template	ICBM 152 Nonlinear Asymmetrical template (2009c) and fsaverage6 surface template.
Noise and artifact removal	Motion correction via MCFLIRT<br>Slice-timing correction via AFNI 3dTshift<br>“Fieldmap-less” distortion correction (Huntenburg et al., 2014)<br>CompCor (aCompCor, tCompCor) noise regressors<br>High-pass filtering (0.008 Hz cutoff)<br>Regression of CSF, WM, and motion parameters
Volume censoring	No volume deletion; motion regressors were included as nuisance covariates.

Statistical modeling & inference

Model type and settings	Voxel-wise GLMs for object and room regressors; searchlight-based multivariate analyses.<br>Subsequent analyses used multivariate classification (multinomial logistic regression) and correlation-based reliability measures.
Effect(s) tested	Neural stability and distinctiveness of room representations (“room reliability”)<br>Relationship between pre-learning room reliability and post-learning object reinstatement<br>Searchlight-based regressions predicting reliability from room features

Specify type of analysis: ☐ Whole brain ☐ ROI-based ☒ Both

Anatomical location(s)	Our full hippocampus region of interest (ROI) was extracted from a freesurfer subcortical parcellation. This ROI was then split into an anterior portion (y > -20) and posterior portion (y <= -20) in MNI space (Guo et al., 2020; Poppenk et al., 2013; Masis-Obando et al., 2022).
Statistic type for inference (See Eklund et al. 2016)	t-tests and Pearson correlations; nonparametric permutation tests for validation.
Correction	False Discovery Rate (FDR) correction applied at q < 0.05 (q < 0.001 for feature regressions).

Models & analysis

n/a	Involved in the study
☒	☐ Functional and/or effective connectivity
☐	☒ Graph analysis
☐	☒ Multivariate modeling or predictive analysis

Graph analysis	Conducted on room adjacency structure (degree, betweenness, closeness, pagerank) from VR environment
Multivariate modeling and predictive analysis	Multinomial logistic classifiers (leave-one-participant-out cross-validation) and correlation-based predictions linking room reliability to object reinstatement
